# Measurement of metabolic activity by telemetric temperature sensing after immunotherapy and chemotherapy on three different mouse tumor models

**DOI:** 10.1186/s12885-026-16308-4

**Published:** 2026-06-26

**Authors:** Sina Meisamy, Kurt Brattain, Qi Shao, Daniel Steinberger, Cade Moffitt, Reza Meisamy, Michael Nelson

**Affiliations:** 1 Breast-Med Inc, Plymouth, MN USA; 2 Geissler Corporation, Minneapolis, MN USA; 3 Department of Neurosurgery, University of Minnesota, Minneapolis, MN USA; 4 Department of Radiology, University of Minnesota, Minneapolis, MN USA; 5 University of Minnesota, Carlson School of Management, Minneapolis, MN USA; 6 The University of Texas at Austin, College of Natural Sciences, Austin, TX USA

**Keywords:** Temperature-based monitoring, Implantable temperature sensors, Cancer therapy response, Immunotherapy, Chemotherapy, Biothermal biomarkers, Thermal profiling in cancer, Mixed-model analysis, Structural equation modeling

## Abstract

This study evaluates within-subject temporal differences between tumor and body temperatures using three cancer models: melanoma, breast cancer, and colon cancer, to monitor the effects of immunotherapy and chemotherapy on temperature differences. In the melanoma arm, TRP-2 immunotherapy led to increased tumor temperatures compared to the control group, with significant overall treatment separation observed at 58 h into the treatment protocol. For breast cancer, AC-Taxol chemotherapy resulted in a drop in body temperature for both treatment and control groups, with significant treatment group separation on tumor vs. body observed at 96 h. In colon cancer, anti-PD-1 immunotherapy showed an upward trend in tumor temperatures, with significant separation from the control group at 94 h. A series of statistical tests, including mixed-model repeated measures and non-parametric tests, revealed significant differences in temperature trends between treatment and control groups for all cancer types. Furthermore, examination of radial smoothing repeated measures models revealed how the clinical application of this technology could be applied. These results suggest that temperature-based monitoring may be an effective tool for assessing therapeutic responses in cancer treatments, highlighting the utility of thermal measurements in evaluating immunotherapy and chemotherapy efficacy.

**Supplementary Information:**

The online version contains supplementary material available at https://doi.org/10.1186/s12885-026-16308-4.

## Background

Cancerous cells are known to cause temperature changes within their local and surrounding environment. This is predominantly due to alterations in both metabolic rate and cellular perfusion. Changes in the temperature of the local cancer environment during treatment may occur much sooner than changes in tumor size. Therefore, it has been postulated that temperature changes in the local tumor environment may reflect early physiologic changes associated with treatment response for various forms of cancer therapy, including primary systemic therapy (neoadjuvant chemotherapy), immunotherapy, and radiation.

There are two methods of measuring physiological temperature: non-contact and direct contact. Non-contact methods, such as thermography used in cancer screening, measure temperature along the surface of the skin rather than the local tumor environment. Conventional contact-based methods, such as thermometers, can only measure temperature on the surface of the human body. Furthermore, other methods, such as MR thermometry and photoacoustic thermometry, can be either too costly or too complex, making them unsuitable for monitoring deep body temperatures over long periods [1, 2]. Therefore, a simpler and more reliable method for measuring the real-time temperature of the local cancer environment is needed to assess early responses to cancer treatment.

Proposed methods for remote temperature monitoring in clinical settings can be performed using telemetric temperature sensors [3, 4]. Similarly, this approach has been utilized in pets and farm animals using implantable microchips that transmit data to an external transponder [5, 6]. However, it should be noted that these small wireless transponders are too large for use in tumor beds [7].

In this paper, we utilized a battery-free microchip small enough to implant in a tumor bed to consistently transmit reliable temperature changes in both the tumor and the basal temperature of the organism. This was conducted using three cancerous animal models (melanoma B16, breast cancer 4T1, and colon cancer MC-38), which underwent treatment with adoptive T cell transfer, AC-Taxol chemotherapy, and anti-PD-1 immunotherapy, respectively. These groups were compared to a control group that did not receive treatment. Tumor temperature and body temperature were measured multiple times per day in both the control and treatment groups. The goal of this study is to provide basal and tumor temperature measurements in cancerous models that exhibit disease progression and tumor size changes compared to those showing an objective response to their treatment regimen. We hypothesize that changes in in-vivo temperature measurements of both the tumor bed and the basal body temperature can help identify an early response to cancer treatment regimens.

## Methods

### Temperature chip placement and measurements

All animals used in this study and the experimental procedure protocol were reviewed and approved by the Institutional Animal Care and Use Committee (IACUC) at the University of Minnesota. All experiments were performed in accordance with relevant guidelines and regulations. All methods are reported in accordance with Animal Research: Reporting of In Vivo Experiments (ARRIVE) guidelines. All animals used in this study were as follows: C57BL/6J mice (female, 8–10 weeks) were obtained from Jackson Laboratory (Bar Harbor, ME) and the BALB/c mice (female, 8–9 weeks) were obtained from Envigo. Mice were individually housed during the temperature recording period to allow uninterrupted temperature data collection.

Two sets of temperatures were collected from each of the tumor-bearing mice by a pair of temperature chips. Each mouse bearing a 4–6 mm subcutaneous tumor on the right flank was anesthetized with Ketamine/Xylazine prior to chip implantation, after which one chip was placed into the center of the tumor, while the other chip was placed in the opposite side of the mouse. Detailed procedural description of the in vivo temperature measurement can be found in [8] and Supplementary Information.

For each subject, the temperature measurement taken approximately 48 h after implantation was used to determine an offset value between the two sensor types. This offset was used to adjust the 400 kHz (basal body) sensor to reflect a zero difference from the Geissler Temperature ASIC™ chip (see Geissler packet insert for additional details), which then allowed for detection of deltas between sensors within subjects, reflective of any new changes after the initial 48 h. This offset method facilitates the use of sensor repeatability levels as a valid limit of difference detection (delta). The offset was applied to all readings for each animal in determining temperature differences between tumor and body in serial measures. This method mitigates inherent differences between sensor device types and baseline differences between subjects, allowing for direct comparisons of group temperature deflections from the starting point based on treatment status. Appropriate central tendencies were applied after distributional analysis (e.g., median or mean).

A ± 0.21 °C threshold was used as the minimum delta for valid detection differences in temperature calculations (between tumor and body) due to the 400 kHz sensor having a repeatability of ± 0.20 °C and the GTATM having a repeatability of ± 0.0085 °C (rounded up to ± 0.01 °C). Thus, 0.20 °C + 0.01 °C = 0.21 °C was used to discern valid difference detection levels for serial temperature monitoring within subjects.

### Temperature senor mechanisms and impact on data

A detailed assessment of the data collected with these technologies [sensors] is provided in a supplemental attachment. Using data captured from devices that are a combination of analog and digital components requires unique considerations and thorough validation of their distribution response characteristics across the temperature exposure range, to meet statistical assumptions required for analysis and modeling. These analyses, detailed in the supplemental attachment, demonstrate that the data from the sensors do adhere to statistical methodology requirements and assumptions to execute the analyses here within. Examination of response data using cumulative distribution curves and statistical simulations to generate sensor inherent variability estimates are described based on each sensor type’s reported repeatability and resolution characteristics. This analysis provided a structure on how to use these sensor types paired together acknowledging and accounting for known performance differences between sensor types, and the impact on the primary outcome data (supplement).

**Fig. 1:**
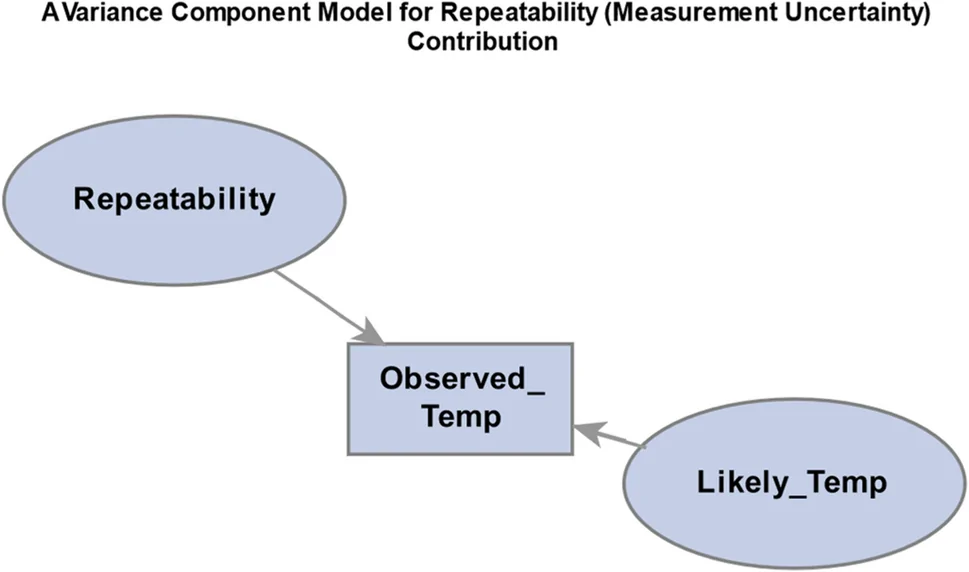
Every observed temperature value results from a likely (by distribution) temperature value

A key component of this study’s data preparation involves the use of a Structural Equation Model (SEM) analysis. The SEM quantified each sensor’s measurement uncertainty by yielding estimates on the variance contribution due to endogenous factors of the sensors. Understanding and quantifying sensor endogenous variance allowed the parsing of variance sources between exogenous and endogenous factors. The SEM approach involves estimating the partial variance generated by sensor’s repeatability (uncertainty) errors. These outcomes were incorporated into the primary outcome measure by defining critical thresholds for differences between Tumor and Body readings. The SEM path analysis can be expressed as follows in Fig. 1.

### Melanoma model and adoptive T cell transfer

Melanoma tumors were established via subcutaneous injection of B16-F10 melanoma cell suspension into the hindlimb flank. Adoptive T cell transfer was delivered to the tumor-bearing mice by intravenous transfer of 1 million tyrosinase-related protein (TRP-2) T cells under brief general anesthesia. The preparation of B16-F10 melanoma cells and TRP-2 T cells are described in [9, 10] and Supplementary Information #1. The transferred antigen-specific cytotoxic T lymphocytes recognize the melanoma epitope as a tumor-associated antigen (TSA) and selectively target B16-F10 cancer cells [10].

### Breast cancer model and chemotherapy

Triple-negative breast cancer (TNBC) tumors were established via subcutaneous injection 4T1 cell suspension into the right hindlimb flank of female BALB/c mice. The preparation of 4T1 breast cancer cells and the establishment of TNBC tumors were described in Supplementary Information #1. Chemotherapeutic cocktail was made with doxorubicin hydrochloride (2 mg/kg), cyclophosphamide (50 mg/kg), and paclitaxel (5 mg/kg) in saline. Adriamycin and cyclophosphamide (AC-Taxol) chemotherapy was administered by intra-tumoral injection of 0.1 mL of the chemotherapeutic cocktail.

### Colon cancer model and anti-PD-1 immunotherapy

The murine colon cancer models were established by subcutaneous injection of MC-38 colon adenocarcinoma cells onto C57BL/6J mice. Immunotherapy was administered by intraperitoneal injection of 100 µg of anti-PD-1 antibody on MC-38 tumor-bearing mice on days 1, 3, and 5. MC-38 tumors are known to be responsive to anti-PD-1 immunotherapy [11]. Technical details of cell culture methods, colon cancer model and its corresponding immunotherapy are described in [8] and Supplementary Information #1.

### Study design

For each cancer model, a cohort of mice was divided into two groups: the control group (the group that did not receive treatment) and the treatment group. Animals were randomized into the control and treatment groups after tumors reached 4–6 mm in diameter.

When necessary, mice were humanely euthanized by Carbon Dioxide (CO_2_) inhalation or Isoflurane overdose; confirmation of death was performed by cervical dislocation. Criteria of euthanasia includes experimental endpoints (experimental plan and tumor endpoint at 16 mm length or 2 cm³) and unrelieved pain or distress (including but not limited to weight loss, inappetence, inability to obtain feed or water, infection, signs of severe organ system dysfunction and other moribund states). Data from mice whose chips were found either dislodged from the tumor or not fully embedded under the skin were excluded from further analysis. Post-mortem X-ray images were taken to visualize the placement of chips in the animals.

### Statistical methodology

All analyses will only include readings that meet the inclusion criteria, starting from the first recorded pairing of tumor and body temperatures after the initiation of the treatment phase, with corresponding control groups analyzed alongside the treatment groups. Our objective is to describe trends in the primary outcomes by treatment group within each study arm.

To evaluate treatment effects on the primary outcome measure, we will employ both non-parametric and parametric statistical methods, selecting parametric tests when the data meets normality assumptions [12–15]. We will specifically utilize a radial smoother [low-rank thin-plate spline] mixed model analysis of variance (ANOVA) with random effects for individual mice, accounting for the influence of treatment group and time on the outcome measures to provide a noise-reduced model that helps highlight clinically relevant patterns of individual subjects [16, 17]. Time points will be recorded with minute-level precision and converted into treatment phase hours, ensuring that we maintain detailed timing in our analysis. While the intervals between readings may vary, they will generally be regular on an 8-hour basis. Reported separation times represent model-based estimates derived from the fitted trajectories and are intended for descriptive interpretation in this exploratory study.

To evaluate treatment effects on the primary outcome measure, we will employ both non-parametric and parametric statistical methods, selecting parametric tests when the data meets normality assumptions. The Kolmogorov–Smirnov Two-Sample Test was used for descriptive comparison of distributional differences between groups and should be interpreted as complementary to visual inspection rather than a primary inferential test, particularly given the repeated-measures structure of the data.

In the radial smoother graphs, we will label each animal’s measurement by a three-group ordinal categorization based on whether the body temperature alone was cooler by at least 0.41 °C, within ± 0.41 °C, or warmer by at least 0.41 °C compared to the baseline body temperature (day 1 of treatment). This threshold of 0.41 °C within an animal is based on the reported repeatability of ± 0.2 °C for the body temperature sensor and 0.41 °C would exceed that level of uncertainty between two measures with the same temperature sensor. This labeling will enable us to visualize individual body temperature changes over time and identify any significant deviations from baseline independent of body vs. tumor temperature differences. Radial smoothing was applied to reduce noise in repeated measurements and to better visualize underlying temporal trends in subject-specific trajectories without overfitting short-term variability. We will test for ordinal level trends with these category labels across treatment groups using the Cochran-Armitage trend test.

### Inclusion / exclusion criteria

The data (3,610 body and tumor temperature readings) from all treatment arms were reviewed, and exclusion criteria were applied similarly. 776 (21.5%) temperature readings occurred before treatment start times and were excluded from the analysis. The primary outcome measurement used in this analysis was the temperature difference between the tumor sensor (134.2 kHz transponder) and the body sensor (400 kHz transponder). As such, only valid pairs where both tumor and body readings (taken within 1 min of each other) were included. There were 4 (0.1%) measures where the difference between tumor and body reading exceeded 6 °C; these were excluded from the analysis. This only occurred in the Melanoma study arm. Once either body or tumor temperature dropped below 30 °C, the remaining data for that subject was excluded from the analysis. This impacted 90 (2.5%) readings in total. Any subject with less than 6 remaining valid readings was excluded from the analysis as some treatments don’t reach peak activity within 5 successive readings. This excluded 6 (8.3%) subjects. The final inclusion count was 66 (91.7%) subjects with 2,668 (73.9%) readings (Tables 1 and 2).

**Table 1:** Inclusion and exclusion summary for subjects and temperature readings by study arm

Study ARM	Readings Prior to Treatment Excluded- *N* (group%)		Tumor vs. Body Reading Difference > 6 °C -Excluded- *N* (group%)		Tumor or Body Readings < 30 °C -Excluded- *N* (group%)		Subjects with < 6 Readings -Excluded- *N* (group%)		Readings Used In Analysis -Included- *N* (group%)		Subjects Used In Analysis -Included- *N* (group%)		
*Breast CA*	**290**	(15.4)	**0**	(0.0)	**32**	(1.7)	**2**	(6.7)	**1550**	(82.5)	**28**	(93.3)	
*Colon CA*	**194**	(27.7)	**0**	(0.0)	**20**	(2.9)	**3**	(18.8)	**432**	(61.7)	**13**	(81.3)	
*Melanoma*	**292**	(28.3)	**4**	(0.4)	**38**	(3.7)	**1**	(3.8)	**686**	(66.5)	**25**	(96.2)	
*[Combined]*	**776**	(21.5)	**4**	(0.1)	**90**	(2.5)	**6**	(8.3)	**2668**	(73.9)	**66**	(91.7)	

**Table 2:** Distribution of subjects and temperature readings across treatment groups by study arm

Treatment Groups by Study ARM		Subjects -Mice- *N* (%)		Temperature Readings *N* (%)	
Breast CA	**Breast CA [Control]**	**16**	(57.1)	**1176**	(75.9)
	**Breast CA [AC-Taxol]**	**12**	(42.9)	**374**	(24.1)
	**Total (study %)**	**28**	(42.4)	**1550**	(58.1)
Colon CA	**Colon CA [Control]**	**6**	(46.2)	**206**	(47.7)
	**Colon CA [anti PD-1]**	**7**	(53.8)	**226**	(52.3)
	**Total (study %)**	**13**	(19.7)	**432**	(16.2)
Melanoma	**Melanoma [Control]**	**15**	(60.0)	**434**	(63.3)
	**Melanoma [TRP-2]**	**10**	(40.0)	**252**	(36.7)
	**Total (study %)**	**25**	(37.9)	**686**	(25.7)

### Primary outcome measure

The primary outcome measure for this analysis is the difference in temperature readings between the tumor and the body after applying an initial offset value to establish a baseline at the start of the treatment phase. This baseline, or setpoint, for each transponder is determined from readings taken 48 h after implantation, allowing a period for inflammation from the implantation procedure to subside. At the 48-hour mark, the recorded temperature for each sensor is used as the offset, effectively setting the reference point at 0 °C for all subjects and sensors. This ensures that all subjects start from the same reference point, allowing subsequent measures to track deviations from this setpoint to calculate the difference between tumor and body temperatures. This approach highlights the clinical relevance of this technology, as it allows for monitoring based on initial readings rather than absolute temperature values.

The formula for the primary outcome measure is as follows:

$$\begin{aligned}&Primary\,Outcome\,Measure\\&=\left[Tumor\,^{\circ}C_{\left(@Time=48\,hours+X\right)}-Tumor\,^{\circ}C_{\left(@Time=48\,hours\right)}\right]\\&-[Body\,^{\circ}C_{\left(@Time=48\,hours+X\right)}-Body\,^{\circ}C_{\left(@Time=48\,hours\right)};\,\\&Where X=time\,in\,the\,treatment\,phase.\end{aligned}$$

This quantity represents the change in tumor–body temperature difference (ΔT) relative to baseline, where positive values indicate that tumor temperature is increasing relative to body temperature over time, and negative values indicate the opposite. From a clinical perspective, this approach allows each subject to serve as its own reference, such that deviations from baseline reflect treatment-associated physiologic changes rather than absolute temperature differences, which may vary across individuals.

All 132 setpoint readings (48 h = 0 °C difference) were excluded from the analyses. In this context, 48 h represents the starting point of the treatment phase, and the repeated measures across time are relative to this treatment timeline. Using this offset and zeroing of tumor vs. body differences provides a reliance only on sensor repeatability and not on sensor accuracy or sensor placement prior to the treatment. In addition, the temperature difference thresholds used in this study are based on sensor performance characteristics and define detectable changes beyond measurement uncertainty, rather than representing clinically established cutoff values.

## Results

### Melanoma and immunotherapy monitoring

The melanoma study arm included 25 subjects who met the inclusion criteria: 60% were in the control group, and 40% were treated with TRP-2 immunotherapy .

**Fig. 2:**
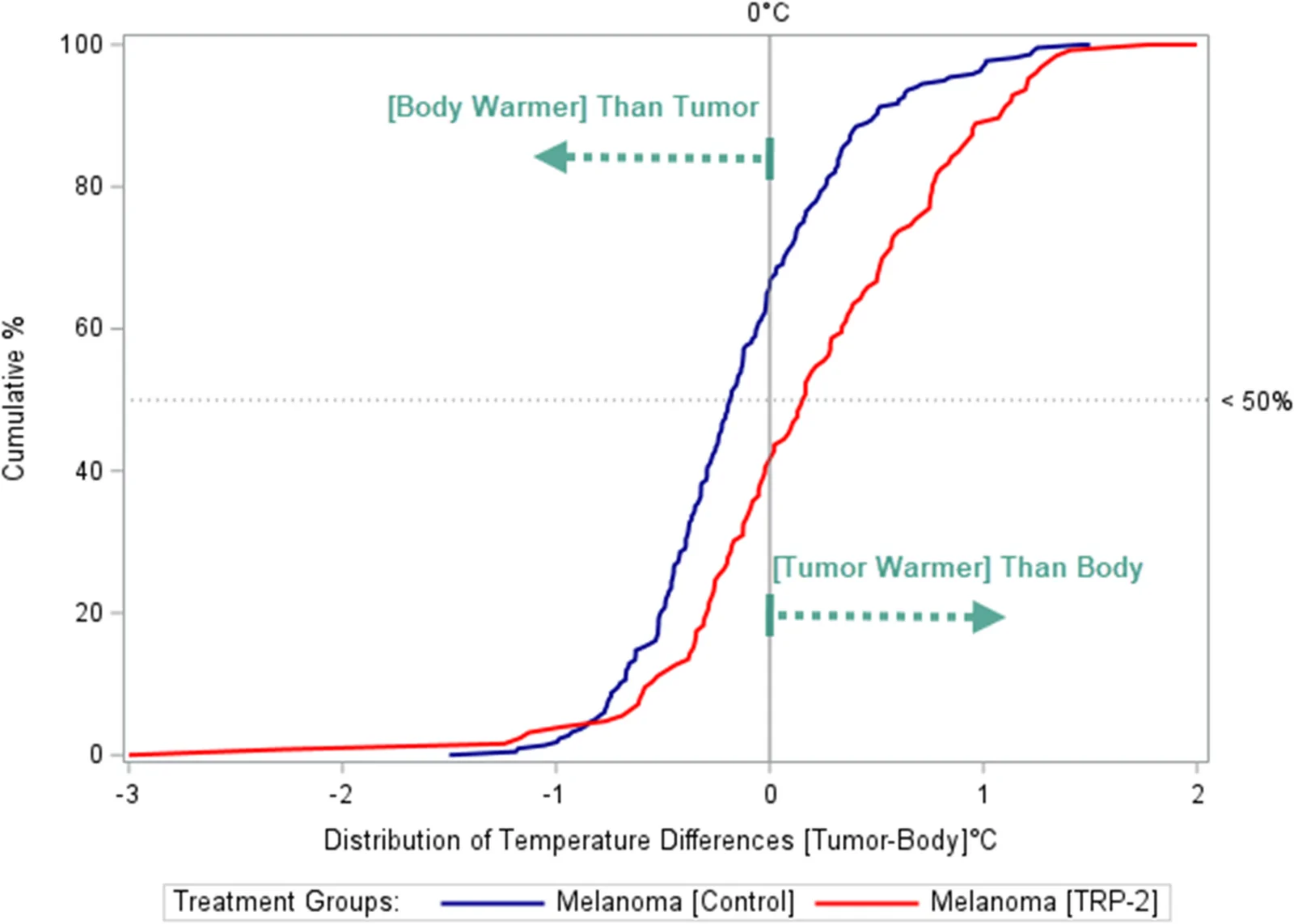
Distributions of temperature differences between melanoma treatment groups

Comparing the distribution of temperature differences [Tumor – Body] between the melanoma treatment groups using a non-parametric Kolmogorov-Smirnov Two-Sample Test yields a KSa of 2.4023 (*p* < 0.0001), rejecting the null hypothesis that the treatment groups come from the same distribution. The [Tumor – Body] differences show a greater number of readings above 0 °C in the TRP-2 group, indicating that tumor temperatures exceeded body temperatures more frequently in this group than in the control group (Fig. 2).

Treatment group analysis was performed with a mixed-model repeated measures model, using subject (mouse) as a covariate, to evaluate the interaction between treatment group and treatment time controlling for subject differences independent of the model main effects and their interactions. The covariate parameter (subject) was significant, with an estimate of 0.2873 °C, Wald-Z = 13.02, *p* < 0.0001, indicating that subject differences were estimated to average 0.29 °C independent of treatment groups and treatment time or interactions. The intercept and treatment group effects were not significant. The interaction between treatment groups and treatment time was significant (F(2,339) = 8.10, *p* = 0.0004). The linear slope for the control group was estimated to be -0.00243 °C/hour (95% CI = [-0.00403, -0.00084]), t(339) = -3.00, *p* = 0.0029. The slope for the treatment group (TRP-2) was estimated to be 0.001967 °C/hour (95% CI = [0.000523, 0.003411]), t(339) = 2.68, *p* = 0.0077. Separation between the treatment groups by more than the 0.21 °C threshold for a valid delta was observed at 58 h, with a temperature difference of 0.2168 °C (95% CI = [0.08546, 0.3481]) higher in the TRP-2 group compared to the control group’s average (t(339) = 3.25, *p* = 0.0013) .

**Fig. 3:**
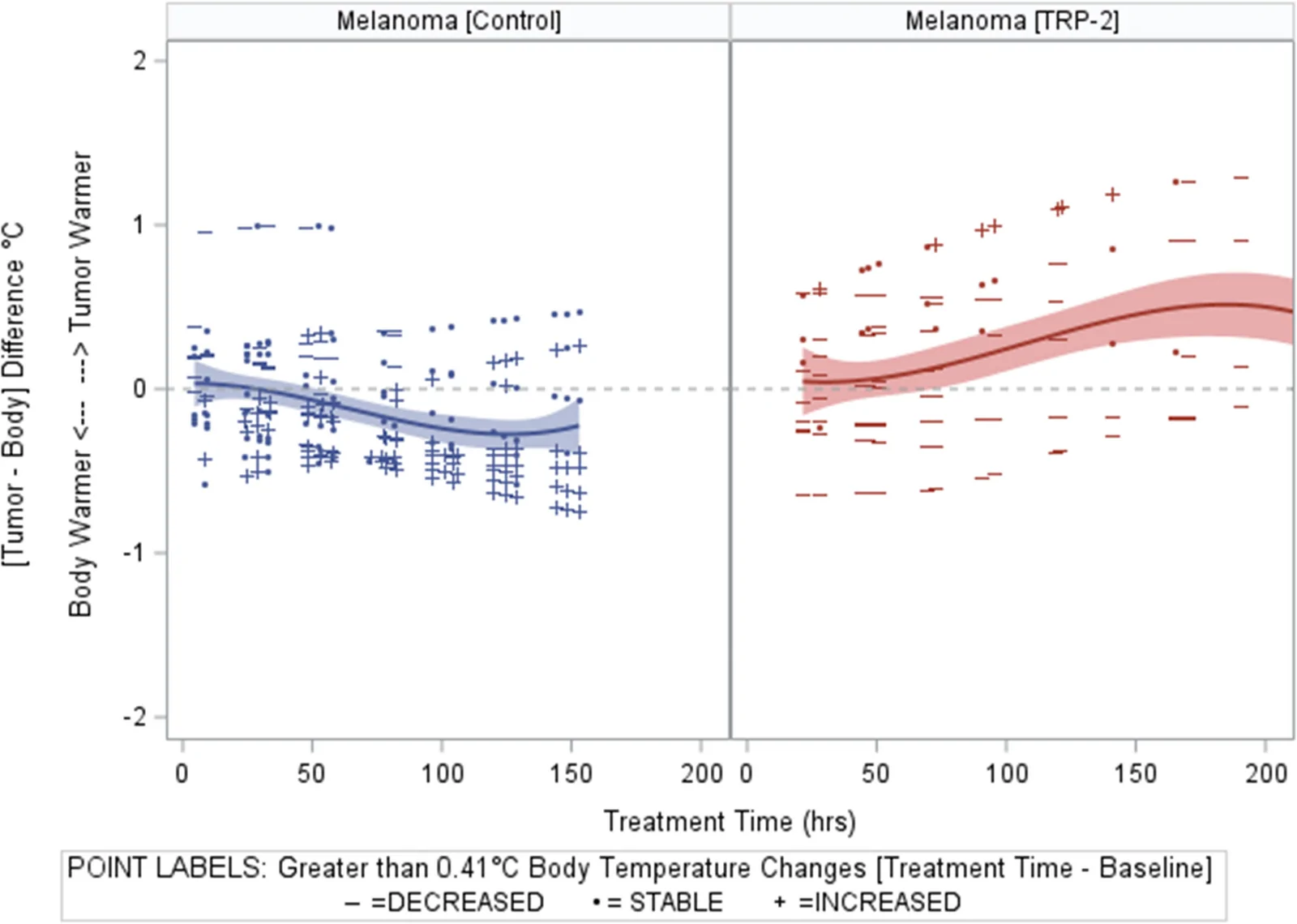
Melanoma - treatment effect radial smoothing graph

A radial smoother mixed model was performed that looked at subject changes over time. The interaction between treatment group and treatment time, with a random intercept, was tested using a type III fixed effect, yielding F(2, 194) = 2.47, *p* = 0.0869. The graph of this model shows, in general, positive slopes for individual subjects in the treatment group (TRP-2) and downward or flat slopes for the control group. For the treatment group, the positive slope trend is observed regardless of which sensor registers the warmer temperature. Body temperature change labeling, comparing body temperatures to baseline values on treatment day #1, visually suggests a difference between treatment groups. A categorical Cochran-Armitage trend test on the ordinal three body temperature categories in reference to treatment day #1 body temperature by treatment group shows a significant trend, with the treatment group having a higher percentage of cooler temperatures compared to day #1 of treatment (Z = -11.1353, *p* < 0.0001) (Fig. 3).

### Breast cancer and chemotherapy monitoring

The Breast Cancer study arm included 28 subjects who met the inclusion criteria. Of these, 57% were assigned to the control group and 43% received the AC-Taxol chemotherapy regimen. The control group remained eligible for inclusion nearly twice as long as the treatment group and accounted for 76% of the temperature readings.

**Fig. 4:**
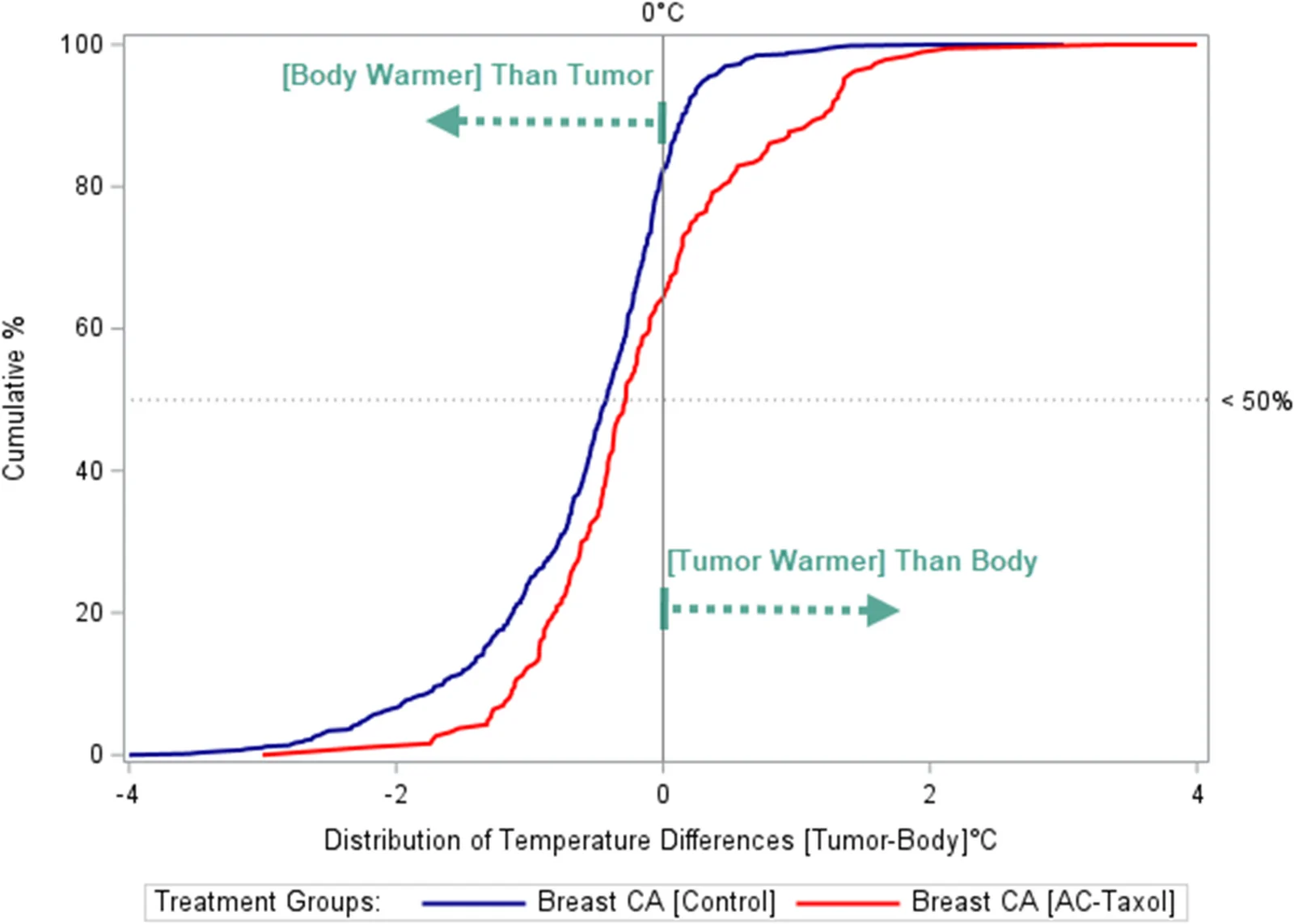
Distributions of temperature differences between breast CA treatment groups

Comparing the distribution of temperature differences [Tumor – Body] between Breast Cancer treatment groups using a non-parametric Kolmogorov-Smirnov Two-Sample Test yields a KSa of 2.3864 (*p* = 0.0005), rejecting the null hypothesis that the treatment groups come from the same distribution. The treatment group’s cumulative distribution is right of the control group’s distribution throughout all percentiles. Individual sensor cumulative plots reveal that the distributions for both the control and AC-Taxol group’s Tumor sensors are nearly identical whereas the Body sensors are generally cooler in the AC-Taxol group compared to that of the control group’s body temperatures (supplemental). This would account for the AC-Taxol [Tumor-Body] differences distribution curve being rightward of the control group curve due to cooler Body temperatures in the face of similar Tumor temperatures (Fig. 4).

Treatment group analysis was performed with a mixed-model repeated measures model, using subject (mouse) as a covariate, to evaluate the interaction between treatment group and treatment time controlling for subject differences independent of the model main effects and their interactions. The covariate estimate (subject) was significant at 0.5028, Wald-Z = 19.63, *p* < 0.0001. The interaction between treatment groups and treatment time was also significant, F(2,771) = 104.29, *p* < 0.0001. The intercept and treatment group effects were not significant. The linear slope for the control group was − 0.00328 °C/hour (95% CI = [-0.00374, -0.00282]), t(771) = -14.09, *p* < 0.0001. The slope for the AC-Taxol treatment group was − 0.00222 °C/hour (95% CI = [-0.00360, -0.00084]), t(771) = -3.16, *p* = 0.0017. The estimated minimum threshold of ± 0.21 °C separation between treatment groups occurred at 96 h of treatment time. At 96 h, the difference was 0.2186 °C (95% CI = [0.0809, 0.3563]) higher in the AC-Taxol group compared to the control group, t(771) = 3.12, *p* = 0.0019. Notably, the AC-Taxol group had fewer readings than the control group (374 versus 1176, respectively). As a result, the 95% confidence interval for the AC-Taxol group is significantly greater than the control group, which should be considered when comparing the regression slopes, as they are nearly parallel.

**Fig. 5:**
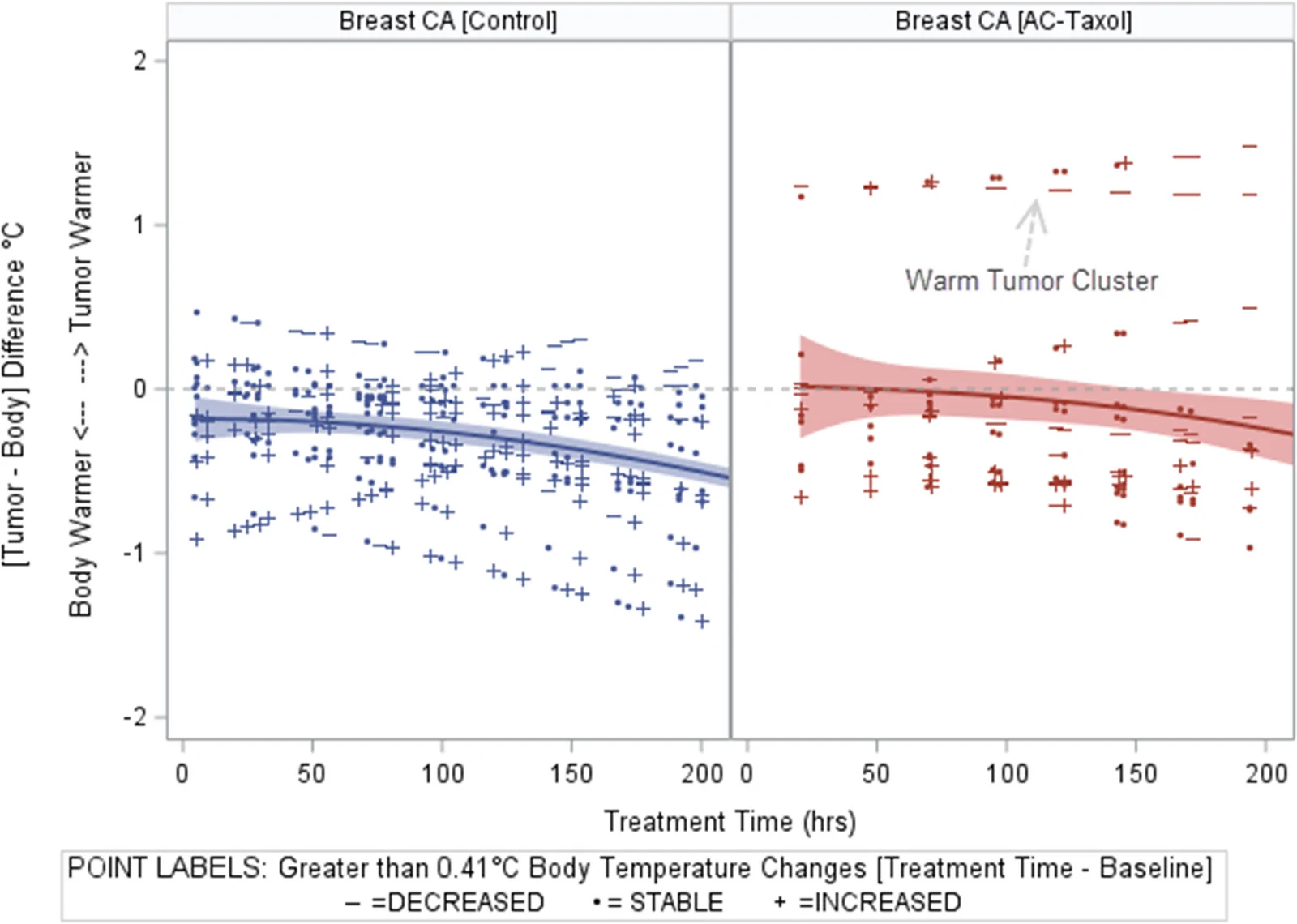
Breast CA - treatment effect radial smoothing graph

The radial smoother mixed model with a random intercept using mice as subjects attempts to delineate individual subject trajectories. The overall interaction between treatment group and treatment time, with the random intercept, yielded a Type III fixed effect test of F(2,512) = 8.91, *p* = 0.0002. Notably, there was a group of mice whose tumor temperatures were at least 1 °C higher than their body temperatures. The labeling of body temperature status categories relative to body temperature at baseline, suggests a difference between the treatment groups. A Cochran-Armitage trend test yielded a significant trend: the treatment group showed a higher percentage of cooler temperatures compared to the baseline (treatment initiation day), Z = 5.8458, *p* < 0.0001 (Fig. 5).

### Colon cancer and immunotherapy monitoring

The Colon Cancer study arm included 13 subjects who met the inclusion criteria. Of these, 46.2% were assigned to the control group, and 53.8% were treated with anti-PD-1 immunotherapy. The temperature readings were distributed in proportion to the number of subjects in each treatment group.

**Fig. 6:**
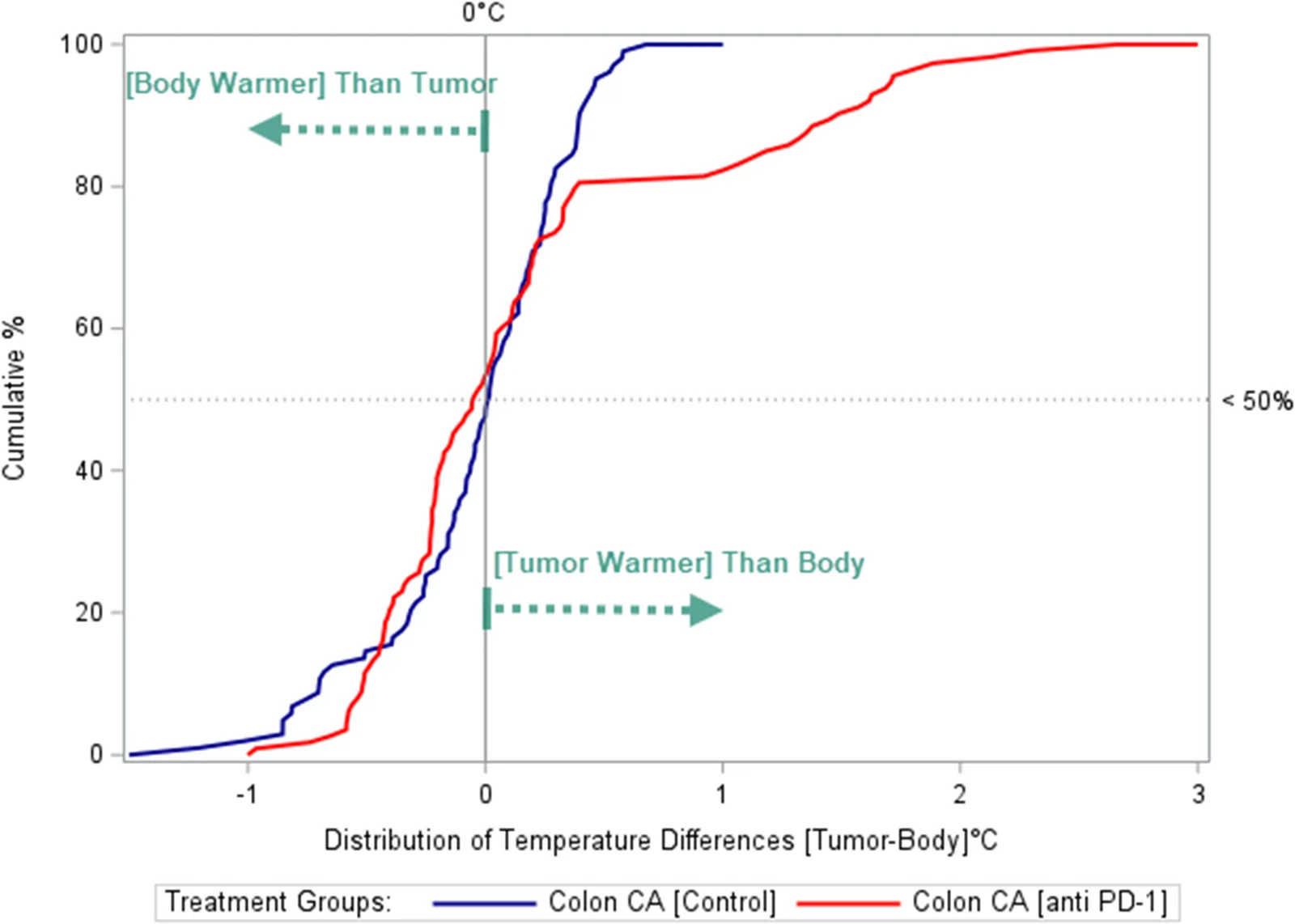
Distributions of temperature differences between colon CA treatment groups

Comparing the distribution of temperature differences [Tumor-Body] between Colon CA treatment groups using a non-parametric Kolmogorov-Smirnov Two-Sample Test yields a KSa of 1.4291 (*p* = 0.0336), rejecting the null hypothesis that the treatment groups come from the same distribution. A significant rightward shift is observed in the treatment group’s cumulative distribution above the 80th percentile (Fig. 6).

A mixed model repeated measures analysis, using the subject (mice) as a covariate, was performed to evaluate the interaction between treatment group and treatment time. The covariate estimate for subject was significant at 0.3140, Wald-Z = 10.30, *p* < 0.0001. The intercept was also significant at -0.3193 (95% CI = [-0.5083, -0.1303]), t(212) = -3.33, *p* = 0.0010. The treatment group effects were not significant. The interaction between treatment groups and treatment time was significant, F(2, 212) = 20.10, *p* < 0.0001. The linear slope for the control group was not significant (0.000779 °C/hour, 95% CI = [-0.00080, 0.00236]), t(212) = 0.97, *p* = 0.3332. In contrast, the slope for the treatment group (anti-PD-1) was significant, with a value of 0.005260 °C/hour (95% CI = [0.00361, 0.00691]), t(212) = 6.27, *p* < 0.0001. Treatment group separation at the minimum ± 0.21 °C threshold was estimated to occur 94 h into the treatment phase. At 94 h, the difference in temperature was 0.2158 °C higher (95% CI = [0.0650, 0.3666]) in the anti-PD-1 group compared to the control group, t(212) = -2.82, *p* = 0.0052. Notably, the anti-PD-1 group exhibited two distinct outcome measure clusters.

**Fig. 7:**
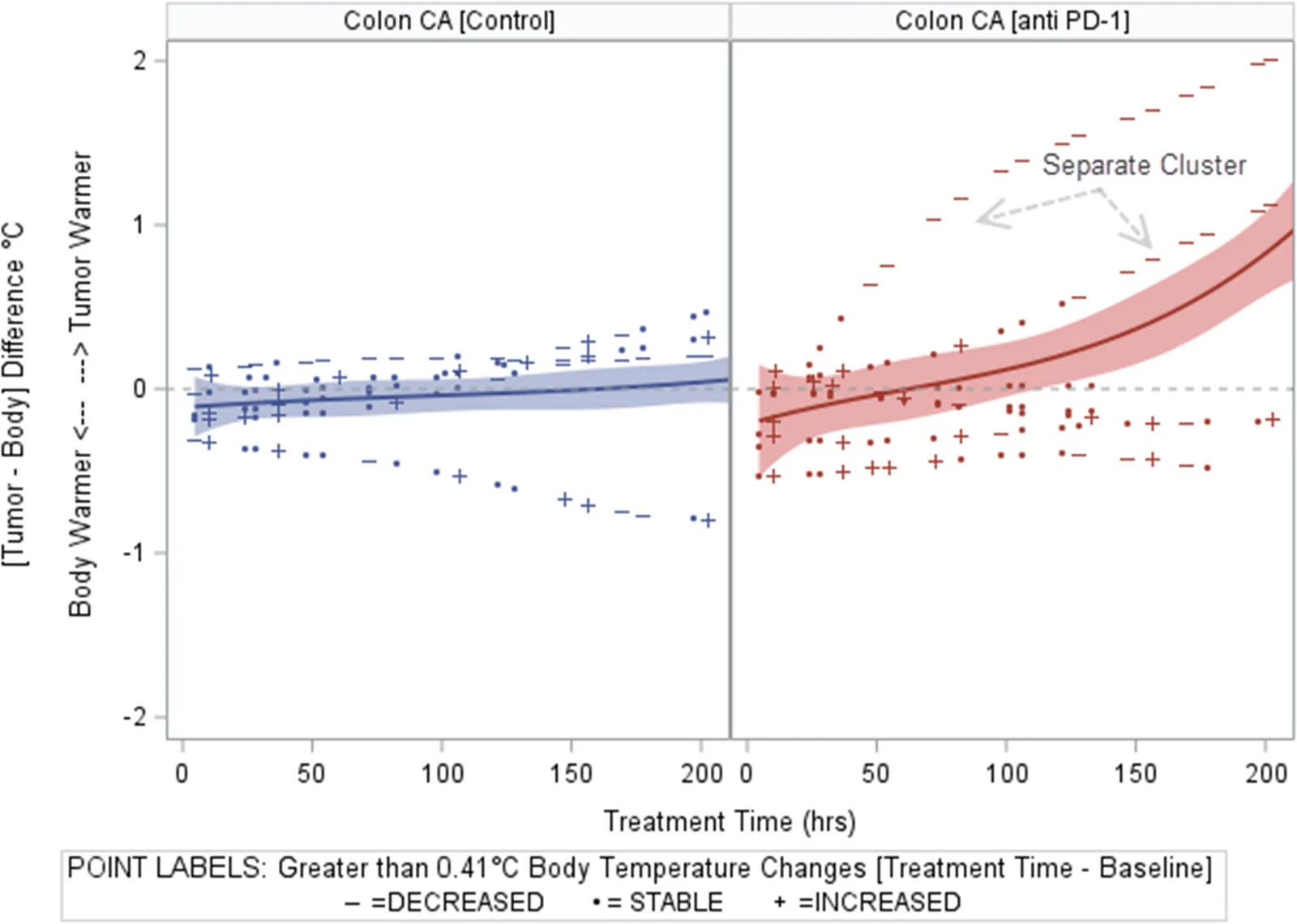
Colon CA - treatment effect radial smoothing graph

A mixed model with random intercept and random radial smoother, using mice as subjects was performed. The overall interaction between treatment group and treatment time, with a random intercept, showed a Type III fixed effect test of F(2, 137) = 1.56, *p* = 0.2134. Notably, a group of mice exhibited an upward trend in outcome measures over time. Interestingly, for these mice, body temperatures were lower than their initial treatment day #1 readings for most of the study. The change in body temperature relative to day #1 was not significantly different between treatment groups. A Cochran-Armitage trend test did not reject the null hypothesis of equal proportions between treatment groups (Z = -0.3015, *p* = 0.7630) (Fig. 7).

## Discussion

Our study explored the temperature dynamics of mice undergoing different treatment regimens, including TRP-2 immunotherapy, AC-Taxol chemotherapy, and anti-PD-1 immunotherapy, compared to control groups. The data suggest that while TRP-2 and AC-Taxol treatments induced temperature changes, the effects were more pronounced in the AC-Taxol and anti-PD-1 groups, with a significant difference in temperature trends over time. Notably, the AC-Taxol group showed an elevated tumor – body temperature distribution curve for the treatment group, likely due to lower body temperatures. The anti-PD-1 group exhibited an upward trend in tumor temperature, with treatment effects becoming minimum threshold significant after approximately 94 h.

Across models, evaluating tumor–body temperature differences enabled differentiation between systemic thermoregulatory effects and localized tumor microenvironment dynamics. Treatments with primarily cytotoxic mechanisms (e.g., AC-Taxol) may influence systemic metabolism and whole-body thermoregulation, potentially attenuating localized tumor-specific heating signals. In contrast, immunomodulatory approaches such as TRP-2 vaccination and anti-PD-1 checkpoint blockade may induce localized inflammatory responses within the tumor microenvironment. Importantly, these temperature shifts are not merely descriptive but reflect underlying biological processes, as changes in tumor–body temperature differentials are consistent with treatment-induced alterations in tumor metabolism, perfusion, and immune activity within the tumor microenvironment.

Immune activation within tumors can promote cytokine release, increased vascular permeability, immune cell infiltration, and enhanced perfusion, all of which may contribute to localized increases in tissue temperature. Similarly, vascular remodeling and metabolic reprogramming within the tumor microenvironment may alter heat production and dissipation dynamics independent of systemic body temperature. Thus, tumor–body divergence provides a relative physiological index that is less sensitive to global temperature shifts and more reflective of tumor-specific biological activity.

Importantly, variability in tumor–body thermal trajectories, particularly within the anti-PD-1 group, may reflect inter-individual differences in immune activation kinetics, baseline tumor vascularization, and treatment responsiveness rather than measurement instability. While these findings suggest that temperature dynamics capture early physiologic changes associated with treatment, direct comparisons with tumor growth kinetics will be required to establish their role as predictive biomarkers.

In the individual sensor distributions, the TRP-2 group showed body temperature shifts, but tumor temperature effects were less clear. This may reflect treatment-induced immune responses or inflammatory changes, suggesting that body temperature could serve as a proxy for immune activity. The AC-Taxol group’s cooler body temperatures may reflect a chemotherapy-induced systemic effect, but the lack of significant tumor temperature changes raises the question of whether these treatments are targeting the tumor directly or inducing the expected thermal responses in the tumor microenvironment. Anti-PD-1 treatment, on the other hand, demonstrated a notable upward trend in tumor temperatures, indicating that immune checkpoint inhibition might alter the tumor microenvironment. However, the presence of two distinct outcome measure clusters in this group suggests heterogeneity in treatment responses.

The heterogeneous clustering observed within the anti-PD-1 group likely reflects inter-individual variability in immune activation kinetics rather than measurement instability. Immune checkpoint blockade can produce asynchronous tumor infiltration, cytokine release, and vascular remodeling across subjects, leading to temporally staggered thermal responses. Thus, variability in tumor–body temperature divergence may represent biologically meaningful response heterogeneity rather than statistical dispersion.

The ability to detect changes in tumor temperature has been previously studied in the context of monitoring response to thermotherapy. Temperature change as an objective measurement of response to therapy is an emerging and promising area of study. While the exact pathophysiology of how changes in temperature reflect underlying molecular processes is not fully understood, there are several theories in the literature. For example, tumor metabolic activity has been shown to affect thermal properties, with the metabolic heat production of a tumor being inversely proportional to its doubling volume time [18]. Furthermore, tumor temperature changes in response to treatment have been documented in prior studies. It has been reported that mitochondrial heat generation during apoptosis is four times higher than in the resting state [19, 20].

Additionally, a reduction in tumor temperature has been linked to tumor necrosis and vascular disruption, often resulting from a decrease in metabolism within the tumor microenvironment. Studies have also demonstrated a positive correlation between oxygen saturation and mitochondrial heating rate in tumor tissue [19, 21]. These findings suggest that temperature changes may reflect metabolic and vascular alterations within the tumor, which could provide valuable insights into treatment response, as seen in our study. The temperature thresholds applied in this study are derived from sensor performance characteristics and should be interpreted as defining detectable changes rather than clinically validated cutoff values.

A key strength of this study is the careful correction for sensor precision differences. Both sensor types (400 kHz and 134.2 kHz) had varying repeatability, and our use of structural equation modeling (SEM) to estimate and correct for measurement uncertainty helped mitigate the risk of regression attenuation bias. These methodological advancements enabled more accurate estimates of temperature difference trends and avoided misleading interpretations due to sensor errors. However, it is important to note that despite these corrections, small variations in the data due to sensor uncertainty remain an inherent limitation.

While these findings are promising, some limitations should be considered and discussed. For example, sample sizes, particularly in the Colon Cancer and Breast Cancer arms, were small, which may have reduced the statistical power to detect more subtle differences. Additionally, the lack of significant temperature changes in the Colon Cancer treatment group suggests that further investigation is needed to explore the underlying biological reasons for this absence of effect. In addition, future studies should also focus on longer follow-up periods to assess the persistence of the observed temperature changes and more importantly, their clinical relevance.

The ability to detect tumor response to cancer therapy has been a longstanding goal in oncology. Currently, assessing treatment response relies on methods that are often time-consuming, expensive, and invasive. For instance, carcinoembryonic antigen (CEA) is a widely used tumor biomarker in colorectal cancer (CRC) but suffers from limitations such as variability in specificity and sensitivity, and differences in CEA levels among individual patients [22, 23]. Similarly, imaging with 18 F-fluorodeoxyglucose (FDG) or other radiotracers used in PET/CT scans is another common method for monitoring treatment responses in some cancers, but these techniques involve recurrent radiation exposure and require patients to visit imaging centers, making them less convenient [24].

The variety of available options for measuring treatment response is a result of the diverse nature of cancer itself. Different cancers may involve distinct tissues, which can influence which diagnostic methods are most appropriate for monitoring treatment progress. However, changes in temperature within the tumor microenvironment occur across all types of cancer, as they all exhibit some degree of metabolic activity associated with neoplasia. This presents an exciting opportunity for temperature-sensing technologies such as the devices used in our study to potentially serve as a standardized, non-invasive monitoring tool that may be applicable across multiple tumor types, particularly solid tumors with measurable microenvironmental changes. By continuously measuring temperature changes, these technologies could provide real-time, objective data to assess tumor response to treatment, thus reducing the reliance on invasive and often costly procedures.

The ability for patients to undergo treatment and remote monitoring without the need for repeated hospital visits could significantly reduce the logistical burden on patients, especially for those who experience difficulties in traveling for follow-up appointments. Transportation from home to healthcare centers is a known social determinant of health, and challenges in accessing care contribute to treatment delays or interruptions for thousands of patients [25]. Remote temperature monitoring addresses this social barrier by providing a low-cost, low-stress method for patients to engage with their treatment while remaining at home. By reducing the frequency of in-person visits, this approach may lead to improved treatment adherence and outcomes.

A limitation of the present study is the unequal sample sizes across certain treatment groups, particularly in models such as AC-Taxol where the number of treated subjects was smaller relative to controls. Although linear mixed-effects modeling and confidence interval estimation partially mitigate imbalance-related bias, smaller group sizes may reduce precision and increase variability in effect estimates. Accordingly, reported effect sizes and confidence intervals should be interpreted in the context of these sample size differences. Another limitation of this study is the relatively small and, in some models, imbalanced sample structure, including unequal subjects and longitudinal readings between groups. While mixed-effects models accommodate such data, these imbalances may reduce precision and the robustness of model-derived estimates, including separation timepoints. Accordingly, these estimates should be interpreted as exploratory rather than definitive. An additional limitation to note is the lack of direct correlation between temperature dynamics and tumor growth measurements or molecular biomarkers, which limits the ability to attribute observed thermal changes to specific biological mechanisms. While these findings were consistent across the models studied, further evaluation across a broader range of tumor types will be required to determine the generalizability of this approach. Future studies with larger and more balanced cohorts will help confirm the robustness of observed thermal divergence patterns. Additionally, future studies integrating longitudinal tumor volume assessment and molecular profiling will also be important to more directly link temperature changes to underlying biological processes and treatment response.

## Conclusions

Our study provides valuable insights into the potential role of temperature dynamics as a biomarker for treatment efficacy. Future studies should aim to investigate the mechanisms behind the temperature shifts observed in the TRP-2 and AC-Taxol groups, as these findings may point to novel pathways for therapeutic intervention. Moreover, incorporating larger sample sizes and longitudinal data would help confirm the robustness of these trends and their applicability in clinical settings.

This research contributes to a growing body of evidence suggesting that temperature dynamics could serve as a non-invasive, real-time indicator of early physiologic changes associated with treatment response. If further validated, this approach could help clinicians assess therapy responses more quickly and accurately, potentially leading to better patient outcomes and personalized treatment strategies. The study underscores the potential of temperature-sensing technologies as an emerging tool in oncology, capable of offering a cost-effective, low-risk, and reliable method to potentially monitor tumor response across a wide range of solid tumors, pending further validation.

## Supplementary Information

Supplementary Material 1.

## Data Availability

The datasets used and/or analyzed during the study that support this article will be made available upon request from the corresponding author on reasonable request.

## Abbreviations

AC-Taxol: Adriamycin–Cyclophosphamide-Taxol
ANOVA: Analysis of Variance
anti-PD-1: Anti-Programmed Cell Death Protein-1 antibody
ARRIVE: Animal Research: Reporting of In Vivo Experiments
ASIC: Application–Specific Integrated Circuit
ATCC: American Type Culture Collection
BMDS: Bio Medic Data Systems
CEA: Carcinoembryonic Antigen
CD8+: Cluster of Differentiation 8 positive (cytotoxic T cells)
CI: Confidence Interval
CRC: Colorectal Cancer
DMEM: Dulbecco’s Modified Eagle Medium
dMMR: Deficient Mismatch Repair
F: F–statistic
FBS: Fetal Bovine Serum
FDG: Fluorodeoxyglucose
GTA™: Geissler Temperature ASIC™
HEPES: 4–(2–Hydroxyethyl)–1–piperazineethanesulfonic acid
IACUC: Institutional Animal Care and Use Committee
IL-2: Interleukin–2
IL-12: Interleukin–12
IQR: Interquartile Range
IV: Intravenous
kHz: Kilohertz
KSa: Kolmogorov–Smirnov statistic (two–sample)
MSI-H: Microsatellite Instability–High
PBS: Phosphate–Buffered Saline
PD-1: Programmed Cell Death Protein 1
Pen-Strep: Penicillin–Streptomycin
PET/CT: Positron Emission Tomography / Computed Tomography
RF: Radio Frequency
RPMI-1640: Roswell Park Memorial Institute Medium 1640
SEM: Structural Equation Modeling
TNBC: Triple–Negative Breast Cancer
TRP-2: Tyrosinase–Related Protein 2
t: t–statistic
Z: Wald–Z statistic
°C: Degrees Celsius
Δ: Difference (delta)
σ: Standard deviation
