# Supplementary material for: Measurement of metabolic activity by telemetric temperature sensing after immunotherapy and chemotherapy on three different mouse tumor models
Source: BMC Cancer. 2026 Jun 26;26:1087. doi: 10.1186/s12885-026-16308-4 (PMC13563789; doi:10.1186/s12885-026-16308-4)
Supplement: Supplementary file 1 [file 12885_2026_16308_MOESM1_ESM.docx]

**Measurement of metabolic activity by telemetric temperature sensing after immunotherapy and chemotherapy on three different mouse tumor models**

**- Supplemental File Part 1 -**

The purpose of this supplemental file (part 1) is to detail the validity of using telemetric temperature sensors with analog and digital components and the extent to which the data from these sensors can be utilized. Additional tables and figures on the study are included for review or in-depth analysis.

**BACKGROUND:**

*Differences between Transponder-Sensors:*

In this study, we utilized two types of transponder sensors to accommodate their proximity within the subjects. Both sensors are powered by an external reader via inductive coupling, allowing for a compact transponder design since all power is drawn from outside the subject. Due to the small size of the subjects, it was necessary for the transponders to operate at different coupling frequencies to ensure accurate transponder identification of each sensor, as the inductive fields are larger than the subjects' bodies. The sensors used were a 400 kHz transponder from Bio Medic Data Systems (BMDS) and a 134.2 kHz Geissler Temperature ASIC (GTA™). The GTA™, being the smaller of the two units, was chosen for implantation in the tumors across all three study arms. These temperature sensors utilize distinct technologies, resulting in significant differences in their precision and repeatability metrics. For this study, repeatability was deemed the most critical performance metric, as the focus was on the temperature differences (deltas) from a setpoint rather than absolute temperature readings. The GTA™ reports a temperature repeatability of ±0.008 °C for the sensor, while BMDS indicates a repeatability of ±0.20 °C of the sensor-reader as a unit.

*Analog to Digital Data:*

The two temperature sensors both use an analog component and a digital component which directly impact the uncertainty of measurements, especially when used in combination as was the case for this study.

Basically, the temperature sensor is an analog device whose temperature determination is converted to a digital value for the purpose of sending a digital packet representation to an external reader. The precision and stability of the temperature surveys are the result of many factors including:

- Calibration accuracy
- The number and range of temperatures used in calibration
- The Analog-to-Digital Converter (ADC) which is deterministic and influenced by:
  - Quantization, Nonlinearity, Jitter, Sampling Rate, Aliasing.

**METHODOLOGY:**

*Determination of Device Uncertainty:*

In the context of these considerations, the 400kHz transponder-sensor system has a repeatability twice that of its digital interval (resolution), namely 0.2°C versus 0.1°C. The 400 kHz sensor reports temperatures in increments of 0.1°C but can change this reporting value by up to 0.2°C even if presented with the exact same environmental temperature. Therefore, the uncertainty in the 400kHz’s measurements is driven by its repeatability of 0.2°C.

The GTA™ transponder-sensor system is a reverse scenario, whereby its repeatability is much smaller than its digital interval, namely 0.008°C versus 0.112°C. These sensors (at calibration) position themselves somewhere in the range of the digital interval 0.001°C – 0.112°C which is their permanent offset position. This offset can be located anywhere within the interval range. It is largely random and forms a [uniform distribution] in combination with other transponders of the same kind. For the GTA™ the repeatability is indicative of a stable and linear ADC system along with a multi temperature point calibration protocol.

What is notable about the GTA™ temperature sensors as a group is that they are uniformly distributed within their digital interval or offset positions. That is, the probability of a sensor having any specific offset value, is the same for all offset values, not the mean or median as an offset. Therefore, even changes in actual temperature that are much smaller than the digital interval distance of 0.112°C will be observed to cause reader changes up or down by a proportion of transponders the same as the temperature change to digital interval width ratio. So, for example in the case of the GTA™, a 0.015°C increase will likely realize a 0.112°C change in temperature readings for 13% of the readers (0.015°C /0.112°C = .13). For a group of 22 transponders, their group average would likely be 0.0153°C on an actual 0.015°C increase even though 3 readers read 0.112°C higher and 19 would read no change. Thus, using averages of many sensors and many readings, we can leverage the group averages to determine the threshold of uncertainty in the GTA™ transponder-sensor system.

A simulation using the following parameters was generated for the GTA™ profile sensor:

- The average number of sensors per study arm (22 mice) was used.
- The average number of readings per sensor (27 readings) was used.
- A temperature range of 6.0°C in 0.025°C step sizes (240 replicates) was used.

The simulation yields an average error of 0.0088°C off actual temperature target. This is in line with the reported repeatability of 0.0085°C. Rounding up, for use as averages would be 0.01°C of uncertainty in the GTA™.

The added average uncertainty for both transponder-sensor types based on repeatability is chosen to be 0.21°C.

*Estimate variance due to device uncertainty:*

Utilizing the level of uncertainty due to repeatability, we now focus on estimating the impact of these uncertainties on data capture. The primary outcome measure will involve combining readings from two types of temperature sensors that have significantly different levels of measurement uncertainty as detailed previously. To address these differences, we will estimate the contribution of measurement errors to the overall variance in the data. This estimate will be achieved using a bootstrapping method that generates 20,000 simulated data sets, incorporating the uncertainty distributions of each sensor type. This step is critical, as neglecting measurement uncertainties could lead to biased conclusions about treatment effects. The theoretical repeatability distribution graphs indicate that the 400 kHz sensor contributes significantly more uncertainty than the GTA™ sensor. In contrast, the GTA™ sensor retains its original reported temperature values. (Figures 1. & 2.)

FIGURE 1: Repeatability Impact Distribution of 400kHz Device.

FIGURE 2: Repeatability Impact Distribution of GTA™ Device.

Using the estimated variances from the bootstrapping data generation, we will apply a Structured Equation Modeling (SEM) path analysis to adjust our data for measurement errors before calculating the primary outcome measures. Structural equation modeling (SEM) offers a method for estimating the inherent variance associated with each sensor type’s repeatability/precision. Each displayed temperature reading can be treated as a value on a distribution, with the most likely value centered around a normal distribution with a mean of 0 and a standard deviation (σ) corresponding to the reported repeatability. By acknowledging the known measurement uncertainties as proxied via reported sensor repeatability, we can enhance our analysis and reduce regression attenuation bias by using these measurement repeatability error distributions.

For each study arm and treatment group, temperature values were assigned a random z-score from a Gaussian distribution with a mean of 0 and a standard deviation based on the sensor's repeatability. Each temperature reading was then adjusted to the center of the repeatability distribution as an estimate of its most probable or likely value, given its observed z-score assignment, resulting in where the z-score of 0 falls within that distribution. Bootstrapping of the values obtained through z-scores was used to estimate the variance between observed and distribution centered values. These estimates are then applied to the SEM structure to approximate the impact each sensor’s uncertainty has on the total variance of the data within the treatment group. The bootstrap resampling process was repeated 20,000 times for all 2,668 readings, with each replicate differing only in the random variations from the repeatability distribution. Finally, the variances for each treatment group and study arm (12 groups in total) were ranked from lowest to highest. The Interquartile Range (IQR) difference in uncertainty variance contribution (75th percentile – 25th percentile) for each group was then used to estimate the average portion of variance attributable to repeatability errors.

A partial variance structural equation modeling (SEM) path analysis was conducted, resulting in adjustments to the raw temperature values based on the model’s estimated error contributions due to measurement uncertainty. Adjustments were not made if the estimated error was less than the reader’s resolution threshold of ±0.1 °C. However, if the adjustments exceeded this threshold, they were applied. The adjusted data was then utilized to establish the offset for the setpoints and to calculate the serial differences between the sensors.

The original observed and adjusted distributions, depicted with Kernel Density Estimation, illustrate how the variability-adjusted data maintains the characteristics of the observed data distribution, albeit with reduced variance. The Normal Density Function distributions clearly demonstrate that the central tendencies of the 400 kHz sensors increase due to the reduction in uncertainty variability. (Figures 3. & 4.)

FIGURE 3: Device Uncertainty Corrections of Observed Readings – Kernel Distribution

FIGURE 4: Device Uncertainty Corrections – Normal Distribution Applicable to Parametric Modeling.

**RESULTS:**

The following sections are additional tables and figures to those in the main manuscript.

*Melanoma and Immunotherapy Monitoring:*

TABLE 1: Melanoma Study Arm Subjects and Readings Counts by Treatment Group

TABLE 2: Melanoma Study Arm 7 Day Average [Tumor – Body] Temperature Differences

| Temperature Comparisons By Treatment Day # | Treatment Group | | | | | |
| --- | --- | --- | --- | --- | --- | --- |
|  | Melanoma [Control] | | | Melanoma [TRP-2] | | |
|  | [Tumor - Body] (°C) | | | [Tumor - Body] (°C) | | |
|  | Mean | 95% | C.I. | Mean | 95% | C.I. |
| Day 1 | **0.000°** | **(-0.15,** | **0.15)** | **-0.045°** | **(-0.24,** | **0.15)** |
| Day 2 | **-0.018°** | **(-0.15,** | **0.11)** | **0.166°** | **(-0.00,** | **0.33)** |
| Day 3 | **-0.050°** | **(-0.24,** | **0.14)** | **-0.009°** | **(-0.27,** | **0.25)** |
| Day 4 | **-0.171°** | **(-0.35,** | **0.00)** | **0.087°** | **(-0.31,** | **0.48)** |
| Day 5 | **-0.292°** | **(-0.46,** | **-0.12)** | **0.401°** | **(-0.13,** | **0.93)** |
| Day 6 | **-0.228°** | **(-0.44,** | **-0.02)** | **0.478°** | **(0.04,** | **0.91)** |
| Day 7 | **-0.248°** | **(-0.56,** | **0.06)** | **0.426°** | **(0.16,** | **0.69)** |

The mean temperatures and 95% confidence intervals (CI) for the first seven treatment days show a generally increasing [tumor – body] temperature trend in the TRP-2 treated group, which was not observed in the control group. (Table 2.)

FIGURE 5: Temperature Change Distribution Analysis for Melanoma Treatment Groups by Body Location.

Cumulative distribution plots of device by treatment group temperature differences from 48 hour zeroing (setpoint) show that the treatment group's body and tumor temperatures shifted leftward (cooler) compared to the control group’s devices temperatures. (Figure 5.)

FIGURE 6: Melanoma Treatment by time graph.

While the model’s confidence intervals separate statistically earlier, the minimum delta threshold of 0.21°C is reached at 58 hours. (Figure 6.)

*Breast CA:*

TABLE 3: Breast CA Study Arm Subjects and Readings Counts by Treatment Group

| Treatment Group | # of Subjects (Mice) N (%) | | # of Temp Readings N (%) | |
| --- | --- | --- | --- | --- |
| Breast CA [Control] | **16** | (57.1) | **1176** | (75.9) |
| Breast CA [AC-Taxol] | **12** | (42.9) | **374** | (24.1) |

TABLE 4: Breast CA Study Arm 7 Day Average [Tumor – Body] Temperature Differences

| Temperature Comparisons By Treatment Day # | Treatment Group | | | | | |
| --- | --- | --- | --- | --- | --- | --- |
|  | Breast CA [Control] | | | Breast CA [AC-Taxol] | | |
|  | [Tumor - Body] (°C) | | | [Tumor - Body] (°C) | | |
|  | Mean | 95% | C.I. | Mean | 95% | C.I. |
| Day 1 | **-0.212°** | **(-0.34,** | **-0.08)** | **0.036°** | **(-0.45,** | **0.52)** |
| Day 2 | **-0.144°** | **(-0.26,** | **-0.02)** | **0.118°** | **(-0.32,** | **0.56)** |
| Day 3 | **-0.156°** | **(-0.28,** | **-0.03)** | **-0.053°** | **(-0.43,** | **0.33)** |
| Day 4 | **-0.333°** | **(-0.45,** | **-0.21)** | **-0.225°** | **(-0.75,** | **0.30)** |
| Day 5 | **-0.273°** | **(-0.38,** | **-0.16)** | **0.051°** | **(-0.23,** | **0.33)** |
| Day 6 | **-0.406°** | **(-0.58,** | **-0.23)** | **-0.166°** | **(-0.49,** | **0.15)** |
| Day 7 | **-0.835°** | **(-0.93,** | **-0.74)** | **-0.287°** | **(-0.50,** | **-0.07)** |

The mean temperatures for the first seven treatment days, along with the 95% confidence intervals (CI), show a sustained negative [tumor – body] temperature difference pattern in the control group, which were not observed in the AC-Taxol treatment group. (Table 4.) The cumulative distribution curves of the device by treatment arm indicate that the AC-Taxol group had a cooler body temperature compared to the control group. (Figure 7.)

FIGURE 7: Temperature Change Distribution Analysis for Breast CA Treatment Groups by Body Location.

Cumulative distribution plots of temperature differences from the setpoint visually show that the AC-Taxol group’s body temperatures shifted leftward (cooler) compared to the control group’s body temperature. 50% of differences in the control group were below 0°C, and 70% of the AC-Taxol group were below 0°C. The tumor temperatures in the treatment group were comparable to those in the control group. This would account for the [Tumor – Body] gradient being higher in the AC-Taxol group compared to the control group. (Figure 8.)

FIGURE 8: Breast CA Treatment by time graph.

*Colon CA:*

TABLE 5: Colon CA Study Arm Subjects and Readings Counts by Treatment Group

| Treatment Group | # of Subjects (Mice) N (%) | | # of Temp Readings N (%) | |
| --- | --- | --- | --- | --- |
| Colon CA [Control] | **6** | (46.2) | **206** | (47.7) |
| Colon CA [anti PD-1] | **7** | (53.8) | **226** | (52.3) |

TABLE 6: Colon CA Study Arm 7 Day Average [Tumor – Body] Temperature Differences

| Temperature Comparisons By Treatment Day # | Treatment Group | | | | | |
| --- | --- | --- | --- | --- | --- | --- |
|  | Colon CA [Control] | | | Colon CA [anti PD-1] | | |
|  | [Tumor - Body] (°C) | | | [Tumor - Body] (°C) | | |
|  | Mean | 95% | C.I. | Mean | 95% | C.I. |
| Day 1 | **-0.106°** | **(-0.24,** | **0.03)** | **-0.194°** | **(-0.33,** | **-0.06)** |
| Day 2 | **-0.062°** | **(-0.19,** | **0.07)** | **-0.118°** | **(-0.29,** | **0.05)** |
| Day 3 | **-0.063°** | **(-0.27,** | **0.15)** | **0.154°** | **(-0.21,** | **0.52)** |
| Day 4 | **-0.275°** | **(-0.57,** | **0.02)** | **-0.094°** | **(-0.46,** | **0.27)** |
| Day 5 | **0.012°** | **(-0.26,** | **0.28)** | **0.177°** | **(-0.19,** | **0.55)** |
| Day 6 | **0.118°** | **(-0.10,** | **0.33)** | **0.150°** | **(-0.28,** | **0.58)** |
| Day 7 | **0.004°** | **(-0.19,** | **0.20)** | **0.710°** | **(0.31,** | **1.11)** |

The mean temperatures for the first seven treatment days, along with their 95% confidence intervals, show that the control group did not exhibit a linear trend during this period. In contrast, the anti-PD-1 treatment group demonstrated an upward trend over time. (Table 6.)

FIGURE 9: Temperature Change Distribution Analysis for Colon CA Treatment Groups by Body Location.

Cumulative distribution plots of temperature differences reveal no significant differences in Body or Tumor temperatures distributions between treatment groups (Figure 9).

FIGURE 10: Colon CA Treatment by time graph.

Here the analysis model demonstrated significant model intercept differences between treatment arms which can be visualized at ~8 hours. While the figure doesn’t show adjusting for this intercept difference (set the intercept for both treatment arms as the midpoint of their difference), doing so would better display that the difference at 94 hours is beyond the minimum threshold separation of 0.21°C difference as determined by device repeatability assessments. (Figure 10.)

FIGURE 11: Colon CA Treatment by time graph – Intercept Adjusted.

This model was adjusted for the significant mixed model’s intercept differences between treatment groups in the Colon CA study by imposing an intercept difference of zero. It’s notable that the slope for the anti PD-1 treatment group is (and was) visually pronounced from that of the control group which is essentially zero. The separation line of > 0.21°C is now at 46 hours. This indicates that the 48 extra hours in the initial model’s time of separation were due to the intercept differences. In consideration of practical application, the intercept adjusted model may be more informative of the utility of this technology. From this data, it’s not quite clear the source of the intercept discrepancy (treatment effect vs. experimental source). (Figure 11.)

**- Supplemental File Part 2 -**

The purpose of this supplemental file (part 2) is to provide additional detail regarding cancer cell preparation, mouse cancer models utilized, adaptive T cell transfer, therapy, and methodology for in vivo temperature measurements.

*Cancer Cell Preparation*

The melanoma cell line (B16-F10) was obtained from the American Type Culture Collection (ATCC). Cells were cultured in Dulbecco's Modified Eagle Medium (DMEM) with 10% FBS and penicillin-streptomycin (Pen-Strep).

The triple-negative breast cancer (TNBC) cell line (4T1) was also obtained from ATCC and cultured in Roswell Park Memorial Institute medium (RPMI-1640) with 10% FBS and Pen-Strep.

The colon adenocarcinoma cell line (MC-38) was obtained from Kerafast, provided by James W. Hodge and Jeffrey Schlom at the National Cancer Institute. Cells were cultured in DMEM with 10% FBS, 2 × 10⁻³ m glutamine, 0.1 × 10⁻³ m nonessential amino acids, 1 × 10⁻³ m sodium pyruvate, 10 × 10⁻³ m HEPES, 50 μg mL⁻¹ gentamycin sulfate, and Pen-Strep.

*Mice Cancer models*

C57BL/6J mice (female, 8–10 weeks) were obtained from Jackson Laboratory (Bar Harbor, ME). BALB/c mice (female, 8–9 weeks) were obtained from Envigo.

When >85% confluence was reached, the cells were detached using 0.05% trypsin-EDTA and resuspended in phosphate-buffered saline at a concentration of 20 million cells mL⁻¹. Tumors were inoculated by injecting 50 μL of cell suspension subcutaneously into the hindlimb of the mice.

Melanoma tumors were established via subcutaneous injection of 50 µL of B16-F10 melanoma cell suspension (20 × 10⁶ cells/mL) into the right hindlimb flank. Tumors reached 4–6 mm in diameter within approximately 9–13 days.

4T1 cells were inoculated on BALB/c mice to model the triple-negative breast cancer (TNBC). Tumors were established via subcutaneous injection of 50 µL of 4T1 cell suspension (20 × 10⁶ cells/mL) into the right hindlimb flank.

MC-38 tumors were established via subcutaneous injection of 50 µL of cell suspension (20 × 10⁶ cells/mL) into the right hindlimb flank. The murine colon cancer model was created by subcutaneous injection of MC-38 colon adenocarcinoma cells onto C57BL/6J mice.

Experiments were performed 9–13 days after tumor seeding when a tumor diameter of 4–6 mm was achieved. All animals were randomized into the control group or the treatment group.

*Adoptive T cell Transfer*

Adoptive T cell transfer for melanoma was achieved through intravenous (IV) transfer of tyrosinase-related protein (TRP-2) T cells, which are cytotoxic (CD8) T cells bearing a genetically encoded high-affinity receptor against the TRP-2 tumor antigen from B16 tumor cells. TRP-2-specific T cells (at least 95% CD8+ Thy1.1+) were isolated using a CD8 T cell isolation kit. The TRP-2 T cells were further stimulated in vitro with interleukin-12 (IL-12) (2.5 ng/mL) and IL-2 (200 U/mL) following established protocols. One million activated TRP-2 T cells were IV transferred to recipient mice through retro-orbital injection under general anesthesia with isoflurane.

*AC-T Chemotherapy*

Adriamycin and cyclophosphamide (AC-T) chemotherapy, which has been used clinically for treating breast cancer, for the 4T1 tumor (TNBC model) was administered by intra-tumoral injection of chemotherapeutic agents (0.1 mL), consisting of doxorubicin hydrochloride (2 mg/kg), cyclophosphamide (50 mg/kg), and paclitaxel (5 mg/kg) in saline.

*Anti-PD-1 immunotherapy*

Anti-programmed cell death protein 1 (anti-PD-1) immunotherapy, which has been approved for treating microsatellite instability-high (MSI-H) and mismatch repair-deficient (dMMR) colorectal cancer, has also been demonstrated to be effective in preclinical colon cancer models, including MC-38. Immunotherapy was delivered by intraperitoneal injection of 100 μg of antibody (InVivo MAb anti-mouse PD-1, Clone RMP1-14 from Bio X Cell) on days 1, 3, and 5 for mice bearing MC-38 tumors.

*In vivo temperature measurement*

All mice had 4–6 mm subcutaneous tumors implanted on the right flank. Two sets of temperatures were collected: the basal temperature, which reflects the body baseline, and the tumor temperature, which indicates the internal temperature of the tumor bed.

Mice were individually caged during the temperature recording period. During the contactless measurements, animals were reached by placing the transponders below the cage without touching it to minimize disturbance to the animals. Information including date, time, basal temperature, and tumor temperature was recorded. Mice were identified by cage number and the chip ID associated with the 134.2 kHz GTA chip.

The basal temperature was measured in the subcutaneous space on the left flank using a temperature chip that is 2 mm in diameter and 12 mm in length and transmits data to an external transponder at 400 kHz. The tumor temperature was measured by placing a Geissler Temperature ASIC™ chip, which is 1.5 mm in diameter and 10 mm in length, in the center of the tumor, transmitting data at 134.2 kHz. Both types of chips consist of two parts on each end: a temperature sensing unit made up of a thermistor and an application-specific integrated circuit (ASIC), and an RF transmitter/receiver unit. A thin (< 0.3 mm) anti-migration sheath covering the temperature-sensing end was used to help immobilize the implants. No impairment of animal mobility or signs of discomfort after chip implantation were observed.

Chip implantation was performed under general anesthesia with ketamine/xylazine. Sterile chips were loaded into a trocar, and the skin surface was cleaned with 70% ethanol before the procedure. During the procedure, a small incision (~2 mm, 5–10 mm away from the measurement side) was made to allow the chip to be placed under the skin. The chips were then gently pushed with the temperature-sensing end facing forward, below the dermis on the left flank or directly into the tumor on the right flank. The incisions were then closed to ensure that the chip remained completely within the body.
